# Supplementary figures and images for: Modeling ROI in chronic disease management: a simulation-based framework integrating patient adherence and policy timing
Source: BMC Public Health. 2025 Dec 19;25:4270. doi: 10.1186/s12889-025-25279-3 (PMC12717686; doi:10.1186/s12889-025-25279-3)

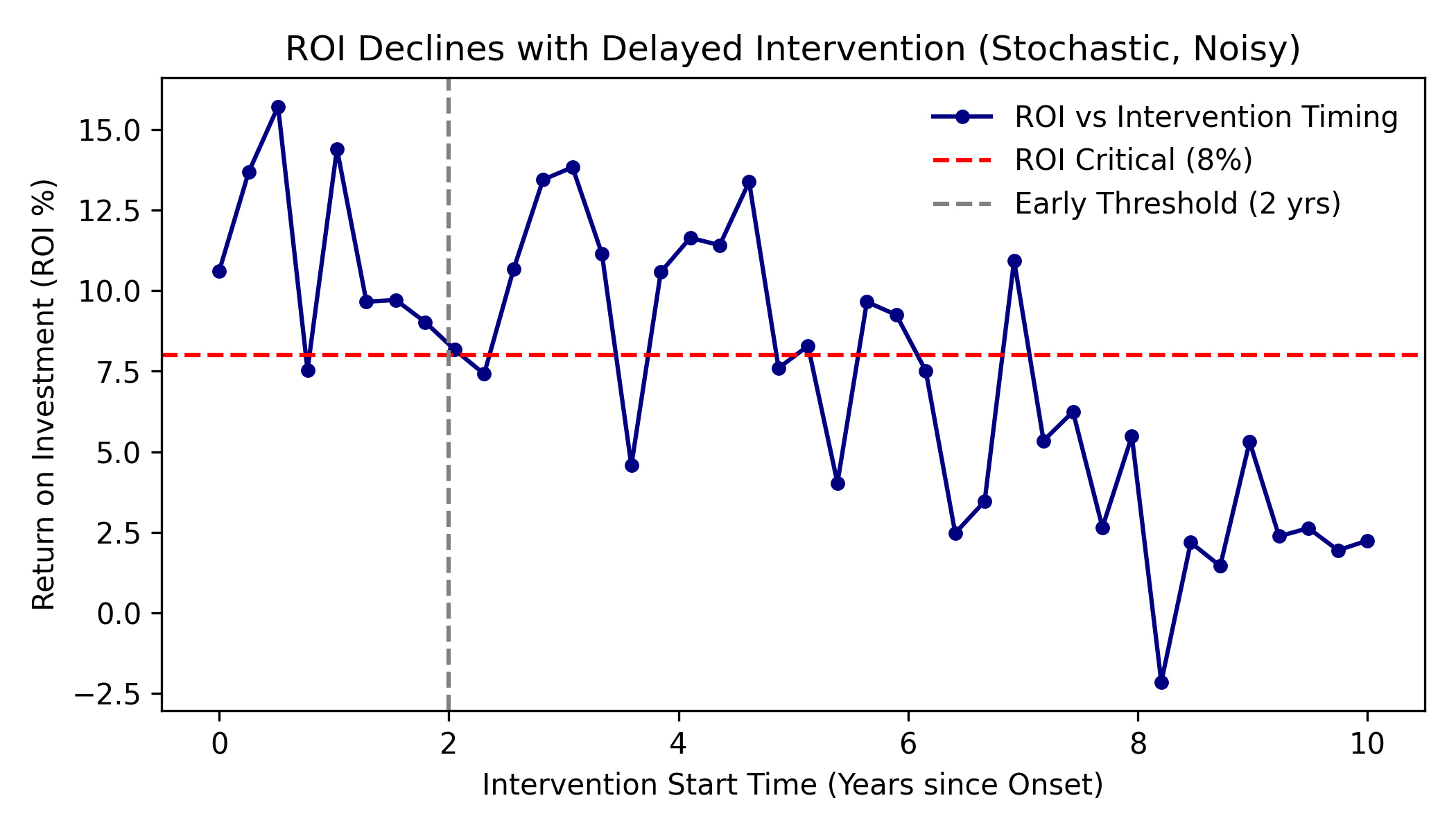

Supplement: Supplementary file 1 — Supplementary Material 1 [file 12889_2025_25279_MOESM1_ESM.zip › FigA1.png]

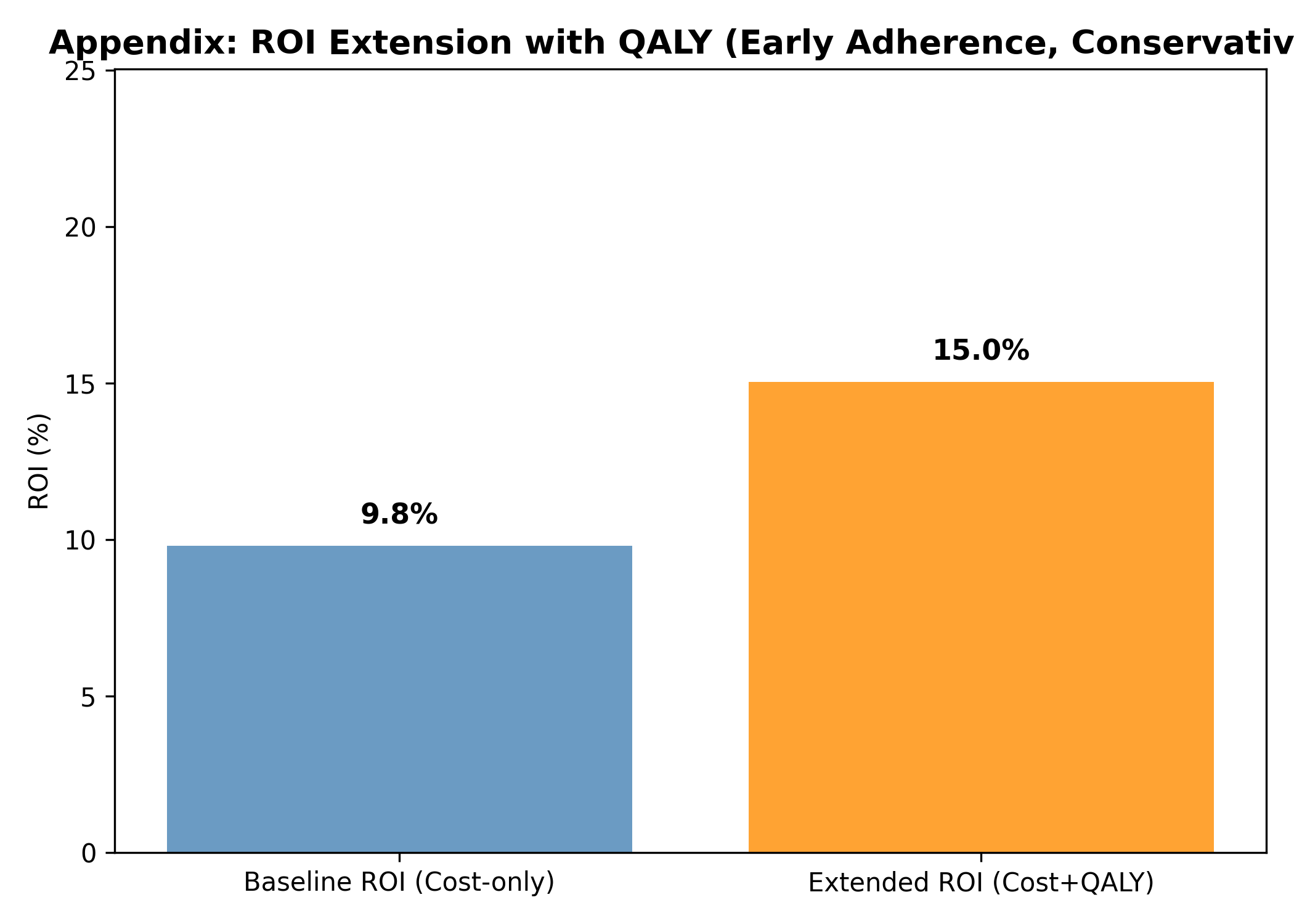

Supplement: Supplementary file 1 — Supplementary Material 1 [file 12889_2025_25279_MOESM1_ESM.zip › FigB1.png]

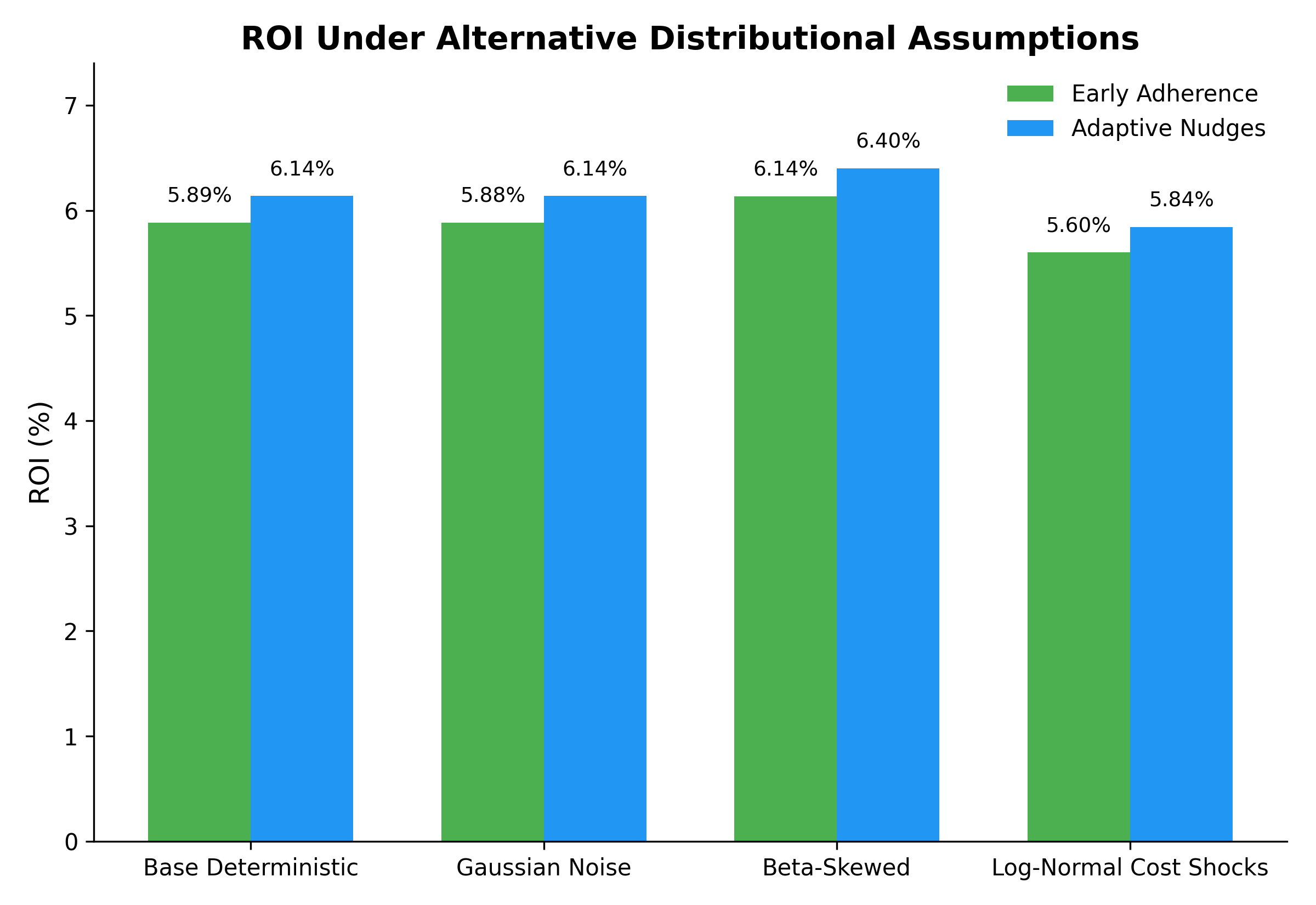

Supplement: Supplementary file 1 — Supplementary Material 1 [file 12889_2025_25279_MOESM1_ESM.zip › FigC1.png]

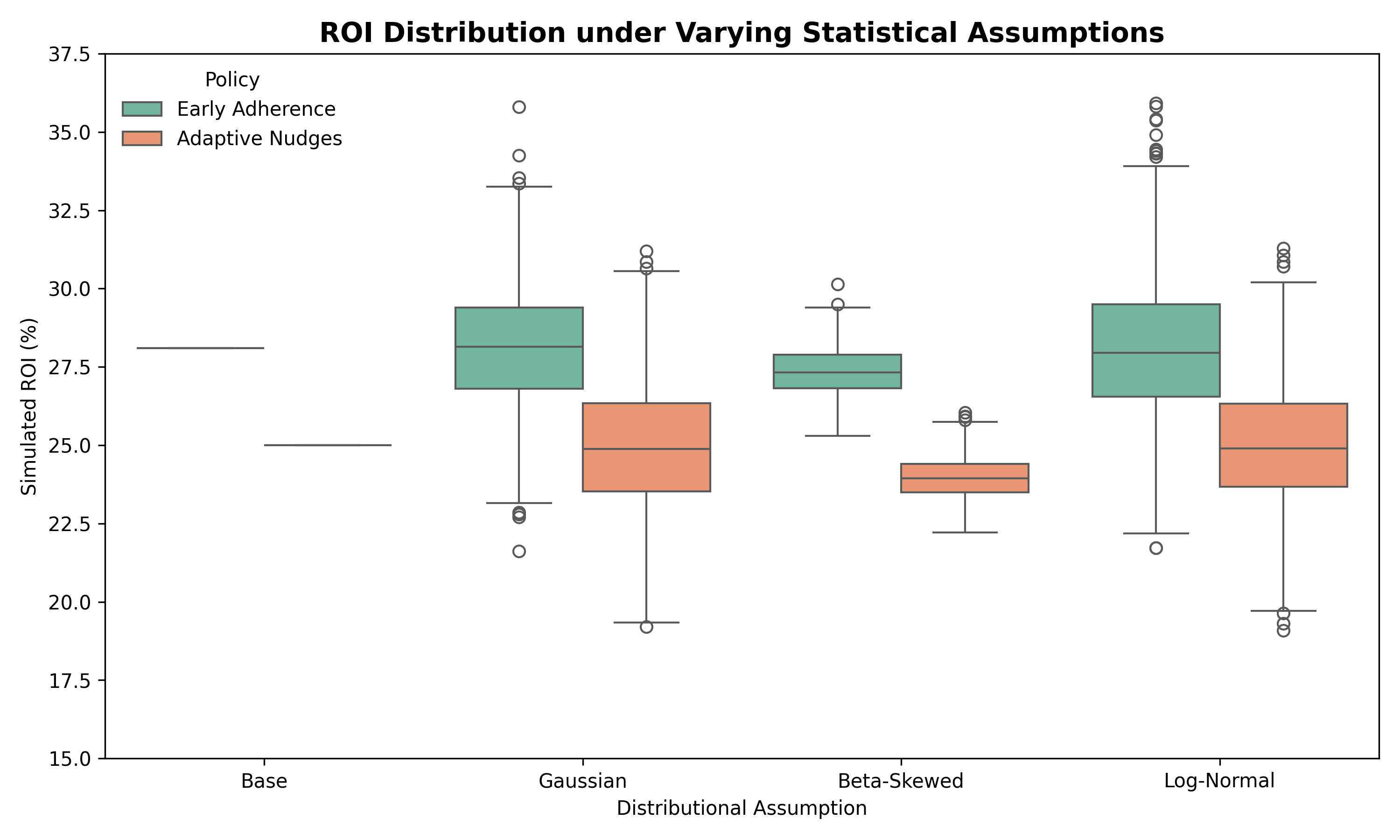

Supplement: Supplementary file 1 — Supplementary Material 1 [file 12889_2025_25279_MOESM1_ESM.zip › FigC2.png]
